# How I manage drainage insufficiency on extracorporeal membrane oxygenation

**DOI:** 10.1186/s13054-020-02870-1

**Published:** 2020-04-15

**Authors:** Bishoy Zakhary, Leen Vercaemst, Phillip Mason, Roberto Lorusso, Daniel Brodie

**Affiliations:** 1 Division of Pulmonary and Critical Care Medicine, Oregon Health and Science University, Portland, OR USA; 2 Department of Perfusion, University Hospital Gasthuisberg, Louvain, Belgium; 3 Department of Surgery, Brooke Army Medical Center, San Antonio, TX USA; 4 Cardio-Thoracic Surgery Department, Heart & Vascular Centre, Maastricht University Medical Centre (MUMC), Cardiovascular Research Institute Maastricht (CARIM), Maastricht, The Netherlands; 5 Columbia University College of Physicians and Surgeons/New York-Presbyterian Hospital, New York, USA; 6 Centre for Acute Respiratory Failure, New York-Presbyterian Hospital, New York, NY USA

**Keywords:** Extracorporeal membrane oxygenation, ECMO, Drainage insufficiency

## Introduction

While extracorporeal membrane oxygenation (ECMO) case volume continues to increase [1, 2], management of patients receiving ECMO remains technically challenging [2, 3]. Iatrogenic injury is a potential contributor to complications and poor outcomes [4–7]. *Drainage insufficiency*, wherein limited pump preload leads to reduced circuit blood flow, is ubiquitous, yet there is no consensus regarding treatment. We propose a physiology-based algorithmic approach to the management of drainage insufficiency.

## Physiology

Analogous to native cardiac physiology, centrifugal pumps are preload dependent such that ECMO blood flow is compromised when there is a mismatch between venous return into the drainage cannula and the drainage pressures at the cannula ports. This relationship is modeled by the Hagen–Poiseuille equation:

$$ \Delta P=8\cdotp Q\cdotp \eta \cdotp L/\pi \cdotp {r}^4 $$

where Δ*P* is the pressure drop across the cannula, *Q* is the blood flow rate, *η* is the blood viscosity, *L* is the cannula length, and *r* is the cannula radius.

For increasing pressure drop along the drainage cannula (Δ*P*), a higher venous return into the drainage cannula is required to maintain equilibrium. When venous return lags behind the pressure drop, drainage insufficiency results. Collapse of the non-rigid vasculature around the drainage ports leads to occlusion of the drainage holes and loss of blood flow. As venous blood reaccumulates, the vasculature returns to its natural state and blood flow resumes.

## Etiology

Drainage insufficiency occurs when there is insufficient venous return or excessively negative drainage pressure.

### Insufficient venous return

Etiologies that reduce venous return into the drainage cannula include hypovolemia, vasodilation, Valsalva maneuver, and inflow obstruction. Venous return may also be dependent on the position of the cannulae and the capacitance of the vasculature containing the drainage ports. For drainage cannulae in the inferior vena cava (IVC), venous return may be compromised by intra-abdominal hypertension (IAH); in the superior vena cava or right atrium, venous return may be compromised by increases in intrathoracic or pericardial pressures.

### Excessively negative drainage pressure

Excessively negative drainage pressure results from too high a pump speed relative to inflow resistance and blood volume. This may occur iatrogenically, when the pump speed is set higher than required, or by necessity, in an effort to augment blood flow in inadequately supported patients. This effect is exacerbated in the setting of concomitant insufficient venous return.

### Timing

Drainage insufficiency occurring soon after ECMO initiation may be due to vasodilation, for instance, as occasionally seen with exposure of the blood to the ECMO circuit [8], or to cannulation issues, such as undersized or malpositioned cannulae or vascular injury. Later in the ECMO course, drainage insufficiency may be seen with agitation, as sedation is lightened, or with volume removal.

## Diagnosis

Drainage insufficiency is overtly present when there is variation of ECMO blood flow in association with reduced pump preload. Clinically, there may be movement of the drainage tubing, a phenomenon variably termed “chatter,” “chugging,” or “kicking.” Drainage pressure measurement may be insightful, but the lack of evidenced-based thresholds limits its utility as a standalone indicator [9]. While stable negative pressures are well tolerated, pressure swings may lead to cavitation and hemolysis [10]. In the absence of these overt indicators, drainage insufficiency can be diagnosed when increasing pump speed does not appreciably increase blood flow.

## Management

**Fig. 1:**
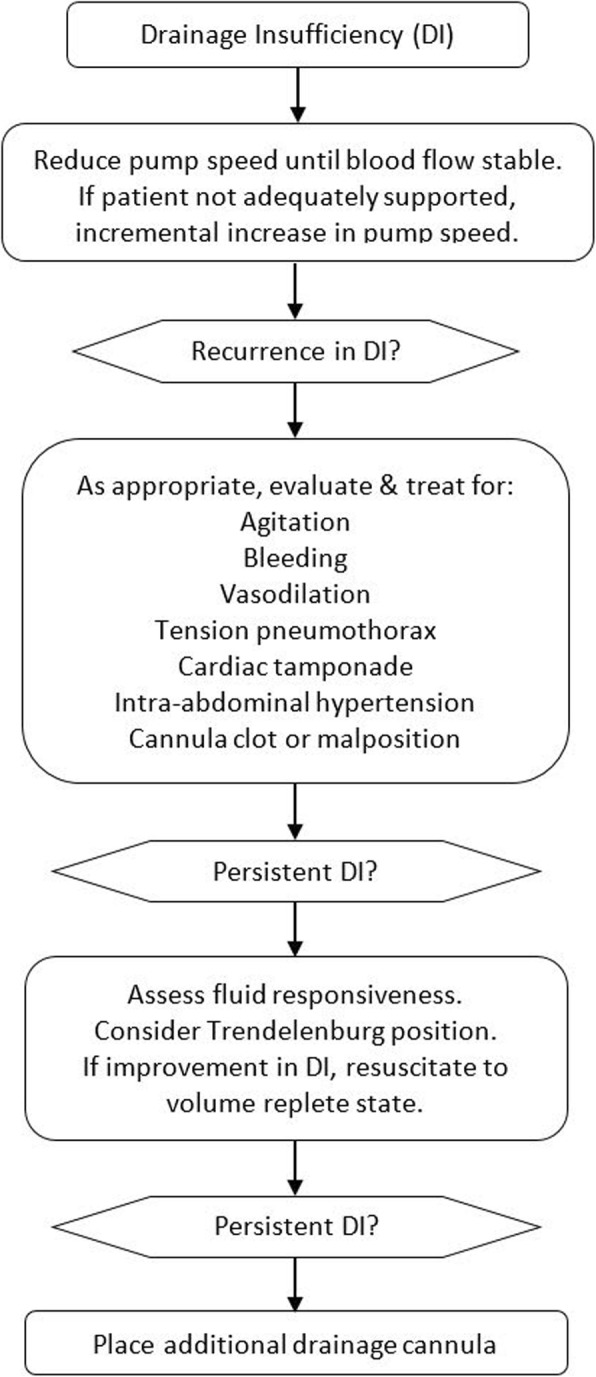
Drainage insufficiency management flowchart. DI drainage insufficiency

Although the differential for drainage insufficiency is broad, most references recommend fluid loading as first-line management [11, 12]. With recent data suggesting positive fluid balance is associated with prolonged ECMO duration and reduced survival [13–15], a more targeted approach may be prudent (Fig. 1).

Management of drainage insufficiency should be aimed at restoring the mismatch between venous return and pressure at the drainage port. As a first step, the pump speed should be reduced until blood flow is stable. If the patient remains adequately supported, the lower pump speed should be maintained; otherwise, an attempt may be made to incrementally increase pump speed while monitoring for recurrence of drainage insufficiency. Of note, the pump speed should be maintained below the level where increases no longer result in higher flow rates.

Clinically evident etiologies for drainage insufficiency should be sought and addressed. For patients exhibiting agitation or coughing, treatment of the underlying cause should be considered; sedation may be required if its risks are outweighed by those of intermittent drainage insufficiency. Inspection of the ECMO circuit from the pump head to the cannula may identify tubing or cannula obstruction. Evaluation for occult bleeding, vasodilation, tension pneumothorax, cardiac tamponade, IAH, and cannula malposition should be performed as clinically appropriate.

For patients with ongoing drainage insufficiency, an assessment of fluid responsiveness should be undertaken. In volume responsive patients, Trendelenburg positioning may resolve drainage insufficiency and should be considered, as a temporizing maneuver, prior to fluid challenge. Subsequent resuscitation should be guided by clinical response, and once a volume replete state is achieved, fluid administration should cease.

If drainage insufficiency persists despite treating clinically evident etiologies and achieving a volume replete state, and assuming no occult cannula thrombosis, then the blood flow requirement is likely greater than can be achieved with the drainage cannula. In this case, placement of an additional drainage cannula should be considered.

## Prevention

Due to the challenges in estimating blood flow requirement prior to ECMO cannulation, we recommend selecting large caliber drainage cannulae relative to the presumed needs of the patient, typically 25 to 29 French for patients with hypoxemic respiratory failure, for instance, to provide the necessary support at low drainage pressures. Femoral drainage cannulae should be inserted sufficiently deep to access the right atrium or intrahepatic vena cava and ensure the proximal drainage holes do not lie in the iliac veins. ECMO support should be titrated to patient needs; as native respiratory or cardiac function recovers, ECMO blood flow should be reduced accordingly, limiting drainage pressures to only what is required, while maintaining a minimum blood flow rate to avoid circuit clotting.

## Conclusion

As the use of ECMO expands, a systematic approach to the management of complex technical issues, such as drainage insufficiency, is essential to improving patient outcomes.

## Data Availability

Not applicable

## References

[1] ELSO (2019). ECLS registry report: international summary.

[2] Brodie D, Slutsky AS, Combes A (2019). Extracorporeal life support for adults with respiratory failure and related indications: a review. JAMA.

[3] Sidebotham D, McGeorge A, McGuinness S, Edwards M, Willcox T, Beca J (2010). Extracorporeal membrane oxygenation for treating severe cardiac and respiratory failure in adults: part 2-technical considerations. J Cardiothorac Vasc Anesth.

[4] Gross-Hardt S, Hesselmann F, Arens J, Steinseifer U, Vercaemst L, Windisch W (2019). Low-flow assessment of current ECMO/ECCO2R rotary blood pumps and the potential effect on hemocompatibility. Crit Care.

[5] Shekar K, Brodie D (2019). Should patients with acute respiratory distress syndrome on venovenous extracorporeal membrane oxygenation have ventilatory support reduced to the lowest tolerable settings?. No Crit Care Med.

[6] Walter JM, Kurihara C, Corbridge TC, Bharat A (2018). Chugging in patients on veno-venous extracorporeal membrane oxygenation: an under-recognized driver of intravenous fluid administration in patients with acute respiratory distress syndrome?. Heart Lung.

[7] Wang S, Chin BJ, Gentile F, Kunselman AR, Palanzo D, Undar A (2016). Potential danger of pre-pump clamping on negative pressure-associated gaseous microemboli generation during extracorporeal life support--an in vitro study. Artif Organs.

[8] Mc IRB, Timpa JG, Kurundkar AR, Holt DW, Kelly DR, Hartman YE (2010). Plasma concentrations of inflammatory cytokines rise rapidly during ECMO-related SIRS due to the release of preformed stores in the intestine. Lab Investig.

[9] Pohlmann JR, Toomasian JM, Hampton CE, Cook KE, Annich GM, Bartlett RH (2009). The relationships between air exposure, negative pressure, and hemolysis. ASAIO J.

[10] Faghih MM, Sharp MK (2019). Modeling and prediction of flow-induced hemolysis: a review. Biomech Model Mechanobiol.

[11] Sidebotham D (2011). Troubleshooting adult ECMO. J Extra Corpor Technol.

[12] Staudacher DL, Bode C, Wengenmayer T (2018). Fluid therapy remains an important cornerstone in the prevention of progressive chugging in extracorporeal membrane oxygenation. Heart Lung.

[13] McCanny P, Smith MW, O'Brien SG, Buscher H, Carton EG (2019). Fluid balance and recovery of native lung function in adult patients supported by venovenous extracorporeal membrane oxygenation and continuous renal replacement therapy. ASAIO J.

[14] Schmidt M, Bailey M, Kelly J, Hodgson C, Cooper DJ, Scheinkestel C (2014). Impact of fluid balance on outcome of adult patients treated with extracorporeal membrane oxygenation. Intensive Care Med.

[15] Besnier E, Boubeche S, Clavier T, Popoff B, Dureuil B, Doguet F, et al. Early positive fluid balance is associated with mortality in patients treated with veno-arterial extra corporeal membrane oxygenation for cardiogenic shock: a retrospective cohort study. Shock. 2020;53(4):426–33. 10.1097/SHK.0000000000001381 31135704

